# FOXO1 Mediates Advanced Glycation End Products Induced Mouse Osteocyte-Like MLO-Y4 Cell Apoptosis and Dysfunctions

**DOI:** 10.1155/2019/6757428

**Published:** 2019-11-25

**Authors:** Citong Zhang, Wei Wei, Minghan Chi, Yao Wan, Xue Li, Manlin Qi, Yanmin Zhou

**Affiliations:** ^1^Department of Oral Implantology, School and Hospital of Stomatology, Jilin University, Changchun, China; ^2^Ministry of Health Key Laboratory of Radiobiology, Jilin University, Changchun, Jilin, China

## Abstract

Osteocyte plays an essential role in bone metabolism by regulating osteoblast and osteoclast activities. Dysfunction or apoptosis of osteocyte will severely endanger the bone homeostasis and result in bone diseases such as osteoporosis. Osteoporosis has been considered as one of the diabetes complications; however, the mechanism is still to be discovered. Advanced glycation end products (AGEs), as the main pathogenic factor of diabetes mellitus, have the capacity to induce osteocyte apoptosis thus sabotaging bone homeostasis. Here, we examined the role of AGE during osteocyte apoptosis and how this effect would affect osteocyte's regulation of osteoblast and osteoclast. Mouse osteocyte-like MLO-Y4 cells were used to study the properties of osteocyte and to examine its biological and pathological function. MTT assay and Annexin V assay showed that AGE significantly induce MLO-Y4 cell apoptosis. qPCR and Western blot results have shown that AGE upregulates proapoptotic gene p53 and its downstream target gene Bax, which leads to enhanced activation of caspase-3, thus inducing apoptosis in MLO-Y4 cells. Increased expression of sclerostin and RANKL in osteocytes has shown that AGE induces osteocyte dysfunction thus severely damaging the bone homeostasis by decreasing osteoblast and increasing osteoclast activities. Furthermore, the role of the transcription factor FOXO1, which is intensely associated with apoptosis, has been determined. Western blot has shown that AGE significantly decreases Akt activities. Immunofluorescence has shown that AGE promotes FOXO1 nuclei localization and enhances FOXO1 expression. Silencing of FOXO1 suppressed AGE-enhanced apoptosis; mRNA and protein expressions of cleaved caspase-3, sclerostin, and RANKL were downregulated as well. Moreover, exogenous FOXO1 increased caspase-3 mRNA levels and caspase-3 transcriptional activity. Lastly, ChIP assay has established the capacity of FOXO1 binding directly on the caspase-3, sclerostin, and RANKL promoter region in AGE environment, providing the mechanism of the AGE-induced osteocyte apoptosis and dysfunction. Our results have shown that FOXO1 plays a crucial role in AGE-induced osteocyte dysfunction and apoptosis through its regulation of caspase-3, sclerostin, and RANKL. This study provides new insight into diabetes-enhanced risk of osteoporosis given the critical role of AGE in the pathogenesis of diabetes and the essential part of osteocyte in bone metabolism.

## 1. Introduction

Osteoporosis, increasing dramatically with population aging, has been considered as a systemic skeletal disease characterized by decreased in bone mineral density (BMD) and increased risk of fracture. Fractures induced by osteoporosis severely affect daily life; vertebral and hip fractures may even lead to increased morbidity and mortality [1]. However, the risk is not limited to weight-bearing bone fracture. Osteoporosis also affects oral health by deteriorating alveolar bone quality which leads to tooth loss [2, 3]. It has been acknowledged that osteoporosis promotes the occurrence and development of periodontal disease and affects the integration and stability of dental implants [4]. Diabetes mellitus (DM) is an exceedingly chronic metabolic disorder which affects bone metabolism deleteriously, which frequently coexists with osteoporosis in the elderly [5, 6]. Recent studies have shown that patients with DM have an elevated risk of osteoporotic fractures [7–9]. Nowadays, osteoporosis has been recognized as one of the diabetic complications.

Under physiological conditions, the progression of bone resorption by osteoclast and bone formation by osteoblast are dynamically balanced. Bone tissue is continuously under remodeling for adaptation to mechanical use and calcium homeostasis [10]. Osteocyte, the most abundant cell in the bone tissue, plays a critical role during the bone remodeling process by regulating osteoclast and osteoblast activities [11, 12]. Osteocyte regulates osteoclastogenesis through its secretion of receptor activator of nuclear factor-*κ*B ligand (RANKL), which is the critical regulator for osteoclast differentiation and activation. Other than osteoblasts and bone marrow stromal cells, osteocytes are the primary source of RANKL secretion in the bone [10, 13]. Sclerostin, which is a protein expressed explicitly by osteocyte, inhibits osteoblast activities and decreases bone formation [14]. Thus, it is safe to say that osteocyte acts as an orchestrator of the bone remodeling process. Therefore, dysfunction or apoptosis of osteocyte will harm the bone homeostasis and result in bone diseases. Osteoporosis is the consequence of an imbalanced bone metabolism which is the excessive bone resorption without the corresponding amount of neoformed bone. It has been recently demonstrated that osteocytes are even involved in the occurrence of osteoporosis directly besides its regulation role in osteoblast and osteoclast behavior.

Accelerated accumulation of advanced glycation end products (AGEs) has been considered as a characteristic feature of DM and osteoporosis [15, 16]. Under hyperglycemia and oxidative stress condition, nonenzymatic chemically modified proteins produce AGEs. Recent studies have demonstrated that AGEs mediate the pathogenesis of osteoporosis by impairing osteoblastic development and function [17, 18]. Several works of literature have argued that AGEs induced osteocyte apoptosis directly, increased sclerostin expression, and decreased RANKL expression in osteocyte-like cells [12, 19, 20]. However, the underlying mechanism of AGE-induced osteocyte dysfunction is still under discovery. It has been reported that AGE stimulates fibroblast apoptosis through transcription factor FOXO1, and FOXO1 silencing can rescue this effect [21]. Other shreds of evidence have shown that FOXO1 is highly involved in the pathogenesis and development of DM and mediates bone metabolism by the regulation of RANKL secretion [22–25]. FOXO1 is a member of forkhead box-O (FOXO) transcription factor, which typically regulates gene expression by binding to DNA and modulating transcription [26]. However, it is difficult to predict the impact of FOXO1 since its function is regulated by both epigenetic and posttranslation protein modifications and its downstream targets are modified by the microenvironment [23, 27–29].

Taken together, we hypothesized that AGEs induce osteocyte dysfunction and apoptosis through a FOXO1-dependent mechanism. Results demonstrate that AGEs lead to a significantly increased osteocyte apoptosis through upregulating p53, Bax, and cleaved caspase-3 expressions. Besides inducing osteocyte apoptosis directly, increased expression of sclerostin and RANKL in osteocyte has shown that AGE-induced osteocyte dysfunction leads to decreased osteoblast activities and increased osteoclast activities thus severely damaging the bone homeostasis. AGE significantly suppresses p-Akt expression, translocates FOXO1 from the cytoplasm into the nucleus, and promotes its function as a transcription factor. FOXO1 has the capacity to direct interaction with the caspase-3, sclerostin, and RANKL promoter regions in osteocytes and enhances their transcriptional activity. Conversely, knockdown of FOXO1 rescued the AGE-enhanced apoptosis and dysfunction of osteocyte. Our results have shown for the first time that FOXO1 mediates the AGE-induced osteocyte apoptosis and its dysregulation of osteoblast and osteoclast. Thus, it is possible that FOXO1 plays a crucial role in the pathogenesis development of DM-induced osteoporosis.

## 2. Materials and Methods

### 2.1. Preparation of AGEs

AGEs were prepared as previously described by Okazaki et al. [30]. Briefly, 50 mg/ml bovine serum albumin (BSA; Sigma-Aldrich, St. Louis, MO, USA) was incubated with 0.1 M DL-glyceraldehyde (Sigma-Aldrich) in 0.2 M phosphate buffer (pH 7.4) at a 37°C sterilized incubator for 1 week, then exhaustively dialyzed against phosphate-buffered saline (pH 7.4) for three days. Nonglycated BSA was prepared at the same time, except that no DL-glyceraldehyde was added. Fluorescence strength of AGE solution was detected at an excitation/emission wavelength of 370/440 nm, which is fortyfold higher than the BSA control.

### 2.2. Cell Culture and Treatment

Experiments were performed with mouse osteocyte-like MLO-Y4 cells from Cell Bank (Shanghai Institutes for Biological Sciences, Chinese Academy of Sciences, Shanghai). This cell line enables us to study the properties of osteocytes and to examine its biological and pathological function [31]. MLO-Y4 cells were cultured on type I collagen-coated plates in a-MEM (Gibco, Life Technologies, Carlsbad, CA, USA) supplemented with 10% fetal bovine serum (HyClone, Logan, Utah, USA) and 1% penicillin-streptomycin (Solarbio, Beijing, China). Cells were kept in an incubator with 5% CO_2_ at 37 degrees Celsius, and the culture medium was changed twice per week. MLO-Y4 cells were incubated with (200 *μ*g/ml) AGE for up to three days after seeding; the control group was incubated with the same concentration of unmodified BSA.

### 2.3. MTT Assay

MLO-Y4 cells were seeded in 96-well plates at 1 × 10^3^ cells per well and treated with 200 *μ*g/ml BSA or AGE for up to three days. The effect of AGE treatment on cell apoptosis and viability was determined by 3-(4,5-dimethylthiazol-2-yl)-2,5-diphenyltetrazolium bromide (MTT) assay according to the manufacturer's instructions (Sigma, Ronkonkoma, NY, USA). Data were detected by a spectrophotometer (Bio-Rad) at 0, 24, 48, and 72 hours.

### 2.4. Annexin V-FITC Apoptosis Detection Assay

MLO-Y4 cells were incubated with 200 *μ*g/ml BSA or AGE separately for three days and collected 24 hours after the transfection of siFOXO1 or its relative control siRNA. Cell apoptosis was assessed with Annexin V-FITC apoptosis detection kit I (BD Biosciences) according to the manufacturer's instructions. Subsequently, 10,000 cells per treatment were counted to assess the number of positively stained cells. Cells with both Annexin V and PI positively stained were recognized as apoptotic cells. Data were exhibited as fold change compared with the control group.

### 2.5. Immunofluorescence

Cells were plated in 96-well plates at 1 × 10^3^ cells per well and incubated with 200 *μ*g/ml BSA or AGE separately for three days before experiments. 4% formaldehyde supplemented with 0.5% Triton X-100 were used for fixation. 2% BSA was used for nonspecific blocking followed by the procedure of incubation with primary anti-FOXO1 antibody (Abcam ab39670, Cambridge, MA, US) or nonspecific IgG (I-1000, Vector Laboratories). The biotinylated secondary antibody was used to associate with primary antibody, and the nucleus was stained with DAPI (S2110; Solarbio, Beijing, China). Exposure time was first set up using the threshold of the highest exposure time with no signal in the nonspecific IgG control. Then, all images were captured at 200× the original magnifications using the same exposure time by a fluorescence microscope (Olympus, Japan). Analysis was performed using an Olympus AX-70 analysis system. Percentage of FOXO1 nuclei-positive cells was set by the number of FOXO1 nuclei-positive cells divided by the total number of FOXO1-positive cells.

### 2.6. RNA Isolation, Reverse Transcription, and qPCR

RNA isolation was performed using the TRIzol (Invitrogen) method. PrimeScript RT reagent kit (RR047, TaKaRa Bio, Toyobo Osaka, Japan) was used for reverse transcription. qPCR was performed using TB Green Advantage kit (#639676, Clontech, Toyobo Osaka, Japan) according to the manufacturer's instructions. Specific primers were as follows: p53: 5′-AGTAAAGGCTCTAAAGCTCACCC-3′ (forward) and 5′-GTAAGAGGTCGGCATTGGAAG-3′ (reverse); Bax: 5′-TGAAGACAGGGGCCTTTTTG-3′ (forward) and 5′-AATTCGCCGGAGACACTCG-3′ (reverse); caspase-3: 5′-TGGTGATGAAGGGGTCATTTATG-3′ (forward) and 5′-AATTCGCCGGAGACACTCG-3′ (reverse); sclerostin: 5′-CGGAGAATGGAGGCAGAC-3′ (forward) and 5′-GTCAGGAAGCGGGTGTAGTG-3′ (reverse); and RANKL: 5′-AGGCTGGGCCAAGATCTCTA-3′ (forward) and 5′-GTCTGTAGGTACGCTTCCCG-3′ (reverse). GAPDH was used as a reference gene and its PCR primers included 5′-GAAGGTGAAGGTCGGAGTC-3′ (forward) and 5′-TTCGGCTTTCCAGTCAGACTC-3′ (reverse). qPCR calculation was performed using *∆∆*CT method.

### 2.7. Western Blot

MLO-Y4 cells were harvested and lysed after three days of incubation of AGE or BSA control. Proteins were separated on an SDS/polyacrylamide gel and transferred to a PVDF membrane (Bio-Rad, Hercules, CA). Followed by nonspecific blocking, membranes were then incubated with the primary antibody (GAPDH: sc-32233, Santa Cruz; p53: ab32389, Abcam; Bax: #5023, Cell Signaling Technology; FOXO1: Abcam ab39670; sclerostin: bs-10200R, Bioss; RANKL: bs-0747R, Bioss; caspase-3: #9662, Cell Signaling Technology; p-Akt: #4058, Cell Signaling Technology; and Akt: #9272, Cell Signaling Technology). The membranes were extensively washed and incubated with a horseradish peroxidase-conjugated secondary antibody (Bio-Rad). The antigen-antibody complexes were visualized by West-Q-Chemiluminescent Sub Kit Plus (BioTang, Waltham, MA).

### 2.8. Transfections and Dual Luciferase Reporter Assay

MLO-Y4 cells were plated into 48-well plates with 70% confluence for siRNA transfections which were performed with 10 nM siFOXO1 (GenePharma, Suzhou, China) or scramble (GenePharma, Suzhou, China) control using siRNAFect (Cwbio, Beijing, China) following the manufacturer's instructions. Full culture medium was replaced by OptiMEM (Gibco) medium one hour prior to transfection. Lipofectamine 3000 was used to perform the transfection according to the manufacturer's instruction. Transfection medium was replaced back to full culture medium after 6 hours. Luciferase reporter assay kit from Promega (cat#: E1960) was used for detection. Cotransfection was performed with empty vector and wild-type FOXO1 vector along with luciferase reporter. Vector and reporter were added at a 1 : 1 ratio according to the transfection efficiency test. For normalizing the verification caused by transfection efficiency, relative luciferase expression values were calculated using firefly luciferase activities divided by Renilla activities as the company instructed.

### 2.9. Chromatin Immunoprecipitation

Approximately 1.5 × 10^8^ MLO-Y4 cells were collected after three days of incubation of BSA and AGE. ChIP-IT Express Enzymatic from Active Motif (#53009) was used to perform chromatin immunoprecipitation (ChIP) according to the manufacturer's instructions. FOXO1 (FKHR) antibody (Abcam ab39670) or nonspecific IgG (I-1000, Vector Laboratories) was used for pulldown. DNA was purified using chromatin IP DNA purification kit (#58002, Active Motif) following the company's instructions before endpoint analysis. The caspase-3 promoter region of 560-745 which contains several consensus FOXO1 elements was detected using the following primers: forward: 5′-GTGTACGTCAGTCCCTTACATC-3′ and reverse: 5′-AGACTCTGACTCTGGGAAGT-3′. The RANKL promoter region of 1046-1245 which contains several consensus FOXO1 elements was detected using the following primers: forward: 5′-GATCTCTGAGTTTGAGGTCAGC-3′ and reverse: 5′-GGACCTGAATTTGACCAGAAGA-3′. The sclerostin promoter region of 562-707 which contains several consensus FOXO1 elements was detected using the following primers: forward: 5′-CTGGATTCCGCCTTCTGTAG-3′ and reverse: 5′-GCAGTCAGGCTGTGGTT-3′. Results were quantified as a percentage of input.

### 2.10. Statistical Analysis

Two-way ANOVA with Tukey's post hoc test was performed for experiments with multiple comparisons. Student's *t* test was performed for comparisons between two groups. All error bars are standard error of the mean. Significance was set at *p* < 0.05. Triplicate samples were examined per group, and experiments were repeated with similar results.

## 3. Results

### 3.1. AGE Induces MLO-Y4 Cell Apoptosis and Dysfunction

**Figure 1:**
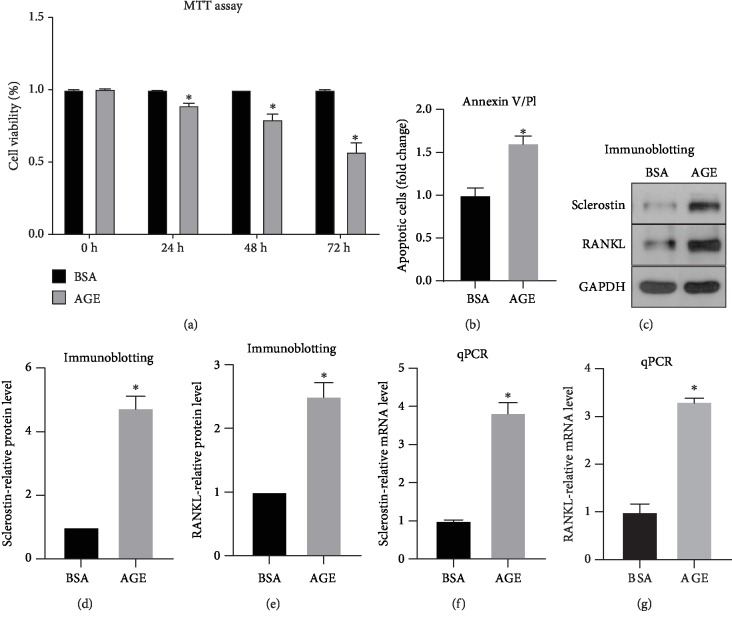
AGE treatment induces MLO-Y4 cell apoptosis and dysfunction. (a) MLO-Y4 cells were incubated for BSA or AGE for up to three days and cell viability was detected by MTT assay at 0 h, 24 h, 48 h, and 72 h. (b) Annexin V/PI assays were performed to detect AGE's role on MLO-Y4 cell apoptosis. Western blot was performed to examine the protein expression of sclerostin and RANKL. (c) Western blot representative images. (d) Quantifications of sclerostin-relative protein expression. (e) Quantifications of RANKL-relative protein expression. qPCR was performed to examine the mRNA expression of sclerostin and RANKL. (f) Quantifications of sclerostin-relative mRNA expression. (g) Quantifications of RANKL-relative mRNA expression. All error bars are standard deviation of the mean. ^∗^*p* < .05 compared to the BSA control group.

We examined the effect of AGE treatment on MLO-Y4 cells. MTT assay was performed to detect cell viability. Cells were incubated with AGE or its negative control BSA for three days. Percentages of cell viability were analyzed at 0, 24, 48, and 72 hours. Results have shown that MLO-Y4 cells' viability gradually reduced by AGE treatment and reached the bottom at 72 hours (Figure 1(a)). This result indicated 72 hours of incubation time as a perfect time point to detect the effect of AGE on osteocyte apoptosis. Annexin V/PI staining was performed to detect AGE's role on apoptosis of MLO-Y4 cells. Result has shown that cell apoptosis exhibits a 1.6-fold increase after incubation of AGE compared with the BSA control group (Figure 1(b)). Furthermore, osteocyte-derived protein sclerostin and RANKL were detected by qPCR and western blot to determine how AGE would affect osteocytes' regulation of osteoblasts and osteoclasts (Figure 1(c)). Western blot results show that AGE leads to a 4.7-fold change in sclerostin expression and a 2.5-fold change in RANKL expression compared with BSA group (Figures 1(d) and 1(e)). qPCR results show that AGE enhances sclerostin expression to 3.8-fold and increases RANKL expression to 3.2-fold compared with BSA group (Figures 1(f) and 1(g)).

### 3.2. AGE Enhances MLO-Y4 Cell Apoptosis by Upregulating p53, Bax, and Caspase-3

**Figure 2:**
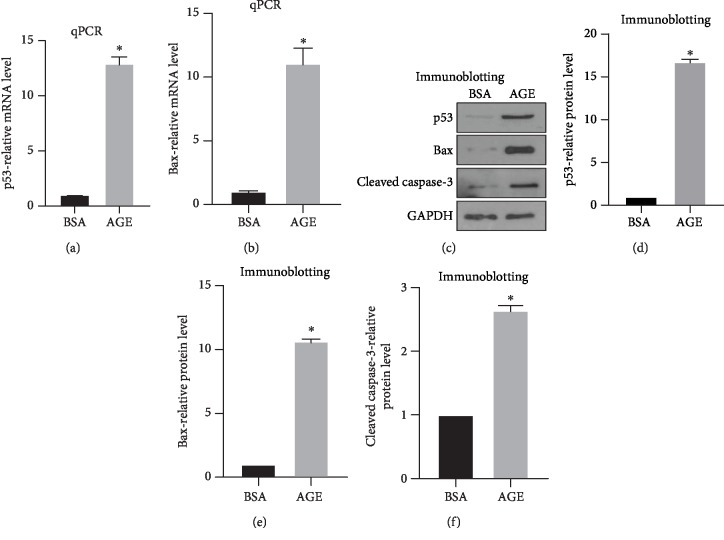
AGE enhances the expression of proapoptotic genes and proteins in MLO-Y4 cells. The mRNA expression of proapoptotic genes p53 and Bax was measured via qPCR. (a) Quantifications of p53-relative mRNA expression. (b) Quantifications of Bax-relative mRNA expression. Western blot was performed to detect p53, Bax, and cleaved caspase-3 expression in protein level. (c) Western blot representative images. (d) Quantifications of cleaved caspase-3-relative expression. (e) Quantifications of p53-relative expression. (f) Quantifications of Bax-relative expression. All error bars are standard deviation of the mean. ^∗^*p* < 0.05 compared to the BSA control group.

mRNA expression of proapoptotic gene p53 and Bax was detected by qPCR after 72 hours of AGE treatment. Quantifications have shown that after AGE treatment, both p53 and Bax mRNA-relative expressions have surged to more than tenfold compared with those in the BSA control group (Figures 2(a) and 2(b)). To verify the mRNA results, Western blot was performed to detect the protein levels of p53 and Bax. Consistent with mRNA results, the protein levels of p53 and Bax were significantly upregulated by AGE (Figure 2(c)). Quantifications have shown that Bax was remarkably increased to elevenfold, while p53 was strikingly increased to sixteenfold when compared with the BSA control group (Figures 2(d) and 2(e)). Cleaved caspase-3 was then detected by Western blot to further determine the apoptosis in MLO-Y4 cells. Result shows that AGE significantly increases cleaved caspase-3 expression (Figure 2(c)). Quantifications have shown that cleaved caspase-3 in the AGE group was twice as much as that in the BSA control group which confirmed AGE-enhanced osteocyte apoptosis (Figure 2(f)).

### 3.3. AGE Enhances FOXO1 Expression and Promotes FOXO1 Nuclei Localization

**Figure 3:**
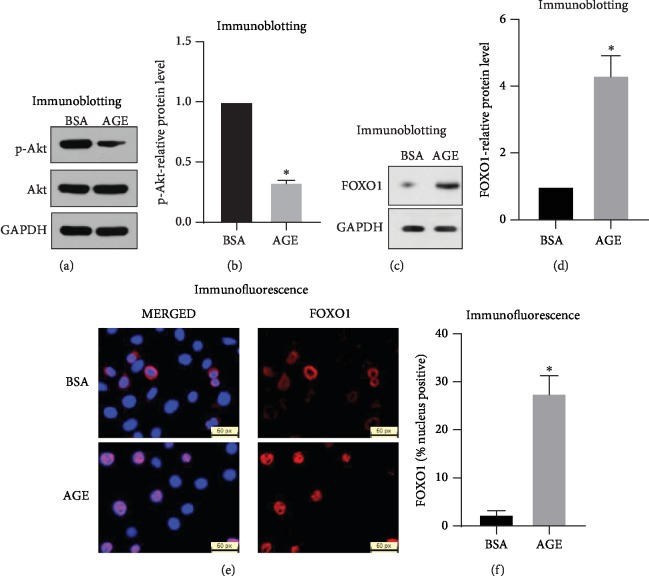
AGE enhances FOXO1 expression and promotes FOXO1 nuclei localization. Western blot was performed to examine the expression of Akt and p-Akt. (a) Akt and p-Akt representative images. (b) Quantifications of p-Akt expression. Western blot was performed to examine the expression of FOXO1. (c) FOXO1 representative images. (d) Quantifications of FOXO1-relative expression. Immunofluorescence pictures taken with FOXO1 antibody to detect FOXO1 nuclei localization, images were taken at 40× for FOXO1-positive cell counting and 100× for exhibiting nuclei localization. (e) Immunofluorescence representative images for FOXO1 nuclei translocation. (f) Percentage of FOXO1 nuclei-positive cells was set by the number of FOXO1 nuclei-positive cells divided by the total number of FOXO1-positive cells. All error bars are standard deviation of the mean. ^∗^*p* < 0.05 compared to the BSA control group.

Akt has been well established to be one of the most important manipulative factors of FOXO1 which induces its phosphorylation and degradation. Akt activities were detected by Western blot using both Akt- and p-Akt- (Ser-473-) specific antibodies. Result has shown that AGE suppresses 70% of p-Akt expression when compared with the BSA control (Figures 3(a) and 3(b)). Western blot was then performed to examine FOXO1 at the protein level. Result has shown that AGE increases FOXO1's expression, and quantification has shown FOXO1 expression was up to fourfold as much as compared with the BSA control group (Figures 3(c) and 3(d)). Immunofluorescence was performed to visualize FOXO1's location after AGE treatment. Images have shown that FOXO1 resides in the cytoplasm in the BSA control group while translocated into the nucleus in the AGE group (Figure 3(e)). Total FOXO1-positive cells and the nucleus FOXO1-positive cells were counted to quantify the percentage of FOXO1 nuclei localization. Quantification has shown that more than 27% of FOXO1 is nuclei positive in the AGE group compared with only 2% in the BSA control group (Figure 3(f)). This result showed that AGE promotes about 25% of FOXO1 translocate from the cytoplasm into the nucleus and functions as a transcription factor. Nuclei localization will clearly lead to an enhanced FOXO1 transcription efficiency and increased the yield of FOXO1 target gene expression.

### 3.4. FOXO1 Deletion Rescued the AGE Enhanced MLO-Y4 Apoptosis and Dysfunction

**Figure 4:**
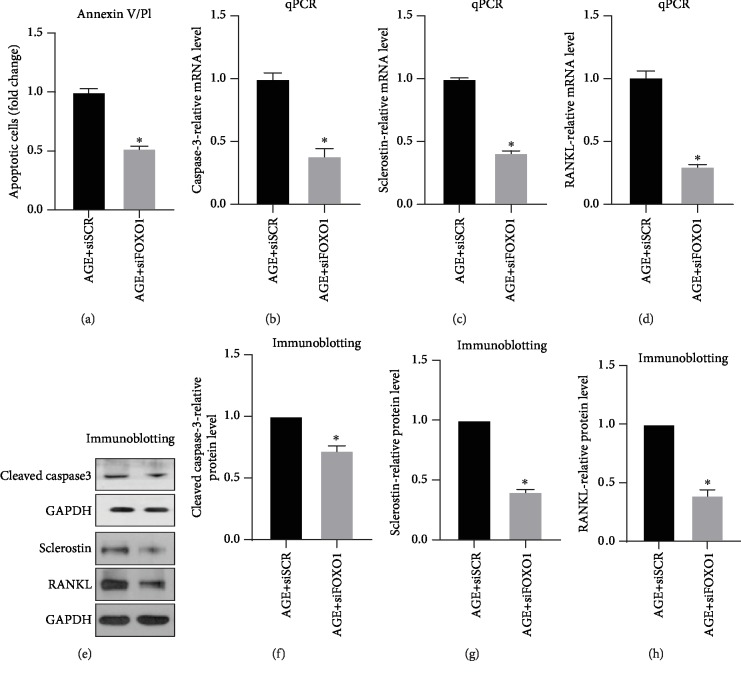
FOXO1 plays as a key regulator of AGE-induced MLO-Y4 cell apoptosis and dysfunction. MLO-Y4 cells were transfected with scramble siRNA or siFOXO1 to detect FOXO1 silencing's effect on AGE-induced MLO-Y4 cell apoptosis and dysfunction. (a) Annexin V/PI assays were performed to detect apoptosis. qPCR was performed to examine the effect of FOXO1 silencing on expression of caspase-3, sclerostin, and RANKL. (b) Quantifications of caspase-3-relative mRNA expression. (c) Quantifications of sclerostin-relative mRNA expression. (d) Quantifications of RANKL-relative mRNA expression. Western blot was performed to detect cleaved caspase-3, sclerostin, and RANKL protein expressions. (e) Western blot representative images (upper GAPDH is for cleaved caspase-3, bottom GAPDH is for sclerostin and RANKL). (f) Quantifications of cleaved caspase-3-relative expression. (g) Quantifications of sclerostin-relative expression. (h) Quantifications of RANKL-relative expression. All error bars are standard deviation of the mean. ^∗^*p* < 0.05 compared to relative scramble siRNA control.

For a better understanding of FOXO1's function during this event, scramble siRNA or siFOXO1 was transfected into MLO-Y4 cells briefly after AGE incubation. Cell apoptosis, mRNA, and protein expression of cleaved caspase-3, sclerostin, and RANKL were detected after transfection. Annexin V/PI staining result has shown that silencing of FOXO1 reduced about 50% of the AGE-enhanced apoptosis (Figure 4(a)). qPCR result demonstrated that 60% of caspase-3 and sclerostin and 70% of RANKL mRNA expressions were reduced by FOXO1 silencing (Figures 4(b), 4(c), and 4(d)). Western blot result demonstrates that the decreased mRNA expression caused by FOXO1 silencing leads to a reduction in protein level (Figure 4(e)). Quantification has shown that siFOXO1 leads to a 30% decrease in cleaved caspase-3, 60% decrease in sclerostin, and 65% in RANKL expression when compared with scramble siRNA in AGE environment (Figures 4(f), 4(g), and 4(h)).

### 3.5. FOXO1 Mediates AGE-Induced MLO-Y4 Cell Apoptosis by Direct Binding to Caspase-3 Promoter Region

**Figure 5:**
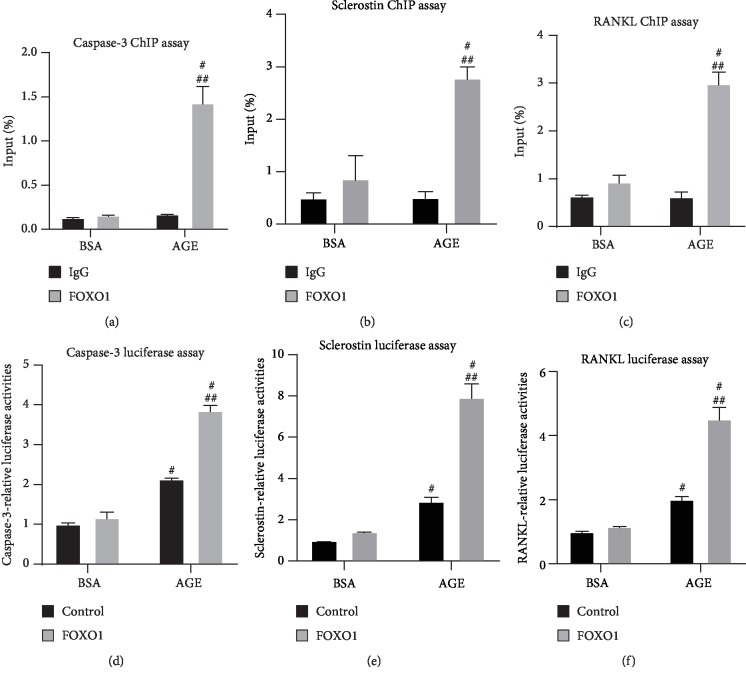
FOXO1 directly binds to the caspase-3, sclerostin, and RANKL promoter regions. ChIP assays were performed in MLO-Y4 cells with pulldown by FOXO1 antibody and PCR amplification of a region of the caspase-3, sclerostin, and RANKL promoter flanking the FOXO1 consensus response element. Results were compared to both its relative control IgG and the FOXO1 binding of the BSA control group. (a) Quantifications of caspase-3 ChIP assay. (b) Quantifications of sclerostin ChIP assay. (c) Quantifications of RANKL ChIP assay. MLO-Y4 cells were cotransfected with empty vector or FOXO1 vector and caspase-3, sclerostin, and RANKL luciferase reporter and Renilla control construct. Results were compared to control (empty vector) and FOXO1 expression plasmid in the BSA control group. (d) Quantifications of caspase-3 luciferase assay. (e) Quantifications of sclerostin luciferase assay. (f) Quantifications of RANKL luciferase assay. All error bars are standard deviation of the mean. ^#^*p* < 0.05 compared to BSA control group. ^##^*p* < 0.05 compared to the BSA FOXO1 group.

To further illustrate the underlying mechanism of this regulation, ChIP assay was performed to determine if FOXO1 is bound directly to the caspase-3, sclerostin, and RANKL promoter regions. Quantifications of caspase-3 results show that in the AGE group, FOXO1 pulldown is eightfold stronger compared with the nonspecific IgG, while no significant difference was found between FOXO1 and IgG in the BSA group (Figure 5(a)). Sclerostin results show that FOXO1 pulldown in AGE group exhibits a 5.5-fold higher compared with the IgG control and RANKL results show a 4.8-fold change (Figures 5(b) and 5(c)). Luciferase assay was then performed to verify the ChIP assay results. Results have shown that exogenous FOXO1 increases caspase-3 twice compared with empty vector in the AGE group but no significant difference was found in the BSA control group, and AGE alone without FOXO1 vector leads to a twofold change in caspase-3 expression (Figure 5(d)). Quantifications show that FOXO1 vector enhances sclerostin expression 2.7-fold and increases RANKL expression twofold compared with empty vector in the AGE group. AGE leads to a 2.9-fold change in sclerostin and a 2.3-fold in RANKL when compared with BSA group (Figures 5(e) and 5(f)). These results are consistent with the ChIP assay results, which FOXO1 only binds to caspase-3, sclerostin, and RANKL promoter regions in the AGE group. Besides that, AGE alone is capable of increases in caspase-3, sclerostin, and RANKL expressions which is consistent with our previous result.

## 4. Discussion

Our study revealed that AGE significantly enhances the expression of p53 and Bax, which results in an increased caspase-3-dependent apoptosis in MLO-Y4 cells. Besides inducing osteocyte apoptosis directly, AGE significantly upregulates osteocyte-derived sclerostin and RANKL indicating that AGE impairs the biological function of osteocytes and leads to inhibited osteoblast activities and enhanced osteoclast activities. These results offered a critical insight that AGE not only induces osteocyte apoptosis but also caused its dysfunction. Furthermore, we provide the foundations for a mechanism as to how these effects were regulated. Akt is an essential manipulative factor of FOXO1 which induces its phosphorylation and degradation. Immunoblotting results have shown that AGE significantly suppresses the activities of Akt, which will lead to upregulated FOXO1 activity. Western blot and immunofluorescence assay verified that AGE upregulates FOXO1 and strengthens its regulation of downstream target genes by translocating FOXO1 into the nucleus. Moreover, silencing of FOXO1 rescued the AGE-enhanced osteocyte apoptosis, sclerostin, and RANKL expressions thus confirming FOXO1's role as a critical regulator during this event. ChIP assay was performed to establish FOXO1's ability to regulate caspase-3, sclerostin, and RANKL expressions through direct promoter binding, and this regulation is verified by luciferase assay. Our results clarified that AGE induces osteocyte apoptosis and dysfunction in a FOXO1-dependent method. These results are critical as they proposed the explanation and possible mechanism for why we are likely seeing increased osteoporosis risk in diabetic patients.

Accumulation of AGE seriously endangers the balance of bone metabolism and results in osteoporosis by increasing osteocyte apoptosis and decreasing osteoblast and osteoclast activities [12, 19, 32]. Our study is aiming to detect the underlying mechanism of how AGE induces osteoporosis and the role of transcription factor FOXO1. Annexin V-FITC apoptosis detection assay results have shown that AGE significantly induces osteocyte apoptosis. Cleaved caspase-3 expression in MLO-Y4 cells in the AGE group was twice as much as that of the control group. These results verified that AGE causes osteocyte apoptosis in a caspase-3-dependent pathway. Furthermore, proapoptotic genes p53 and Bax, which are upper stream signaling of caspase-3, were detected after AGE treatment. Results have shown elevated expressions of p53 and Bax in both mRNA and protein levels as we anticipated. p53-mediated mitochondria intrinsic apoptosis has been intensely studied. It can rapidly translocate from the cytoplasm to the mitochondrial surface upon activation by oxidative stress, leading to apoptosis through interacting with BCL-2 family members, such as inhibiting antiapoptotic gene Bcl-2 and activating proapoptotic gene Bax functions [33–35]. These results suggest that AGE induces osteocyte apoptosis through p53-mediated mitochondria intrinsic pathway which is executed by caspase-3. Other than inducing osteocyte apoptosis, AGE also affects osteocytes' regulation of osteoblast and osteoclast activities. Sclerostin and RANKL expressions were detected, and the results have shown that both cytokines secreted by osteocytes were elevated. The previous study [12] found that AGE2 and AGE3 reduce RANKL expression in the MLO-Y4 cell lysate but upregulate its expression in the supernatant. The main difference between the two studies is that they were using 100 *μ*g/ml of AGEs while we are using 200 *μ*g/ml as a working concentration. This working concentration was decided based on our previous study [36]. 200 *μ*g/ml of AGEs would represent approximately 4.8 nmol/ml of carboxymethyllysine, which approximates the levels of carboxymethyllysine reported in serum (approximately 2.6 nmol/ml). It has been reported that 200 *μ*g/ml of AGEs significantly induce fibroblast apoptosis and increase RANKL expression in chondrocytes [21, 22]. However, it is possible that different levels of AGEs have various abilities to induce RANKL expression. Taken together, our results indicate that AGE induces osteocyte apoptosis and has the capacity to cause osteocyte dysfunction, which would suppress osteoblast activities and enhance osteoclast activities, thus sabotaging bone homeostasis and leads to osteoporosis.

Several studies have revealed that FOXO1 is closely associated with apoptosis. Tumor-necrosis-factor-related apoptosis-inducing ligand (TRAIL) is regulated by FOXO1 and responsible for FOXO1-induced apoptosis [37]. In addition to that, FOXO1 is capable of transactivation of Bim which is a proapoptotic member of the Bcl-2 family that functions in the intrinsic mitochondrial apoptotic pathway [38, 39]. Our study established that FOXO1 mediates AGE-induced osteocyte apoptosis through caspase-3 and promotes AGE-caused osteocyte dysfunction by upregulating sclerostin and RANKL. Silencing of FOXO1 decreases osteocyte apoptosis and cleaved caspase-3, sclerostin, and RANKL expressions indicating that FOXO1 is a key regulator in the AGE-aggravated osteocyte dysfunction and apoptosis, and these reduced expressions are likely due to the direct interaction of FOXO1 with the caspase-3, sclerostin, and RANKL promoter regions that all contain a FOXO1 consensus response element. ChIP assay results have proved that FOXO1 binds to caspase-3, sclerostin, and RANKL promoter regions in the AGE group but not in the BSA control group. This is likely due to AGE significantly suppressing Akt activities, which promotes FOXO1 translocate into the nucleus and increases its transcriptional activities. Indeed, luciferase assay has provided that without exogenous FOXO1, AGE alone induces caspase-3 expression, which could be explained by AGE-induced endogenous FOXO1 nuclei localization. However, this could also be related to FOXO1's posttranscriptional modifications. Phosphorylation has been well known for its regulation of FOXO1's downstream targets [40]. Besides phosphorylation, the previous study has shown that FOXO1's binding activities are heavily linked with its acetylated status [25]. It could be possible that AGE regulates the downstream genes of FOXO1 by altering its posttranscriptional modification. This will become a future interest in our study.

In summary, our experiments show that AGE significantly induced osteocyte apoptosis and caused its dysfunction. AGE upregulates p53 and its downstream target Bax then leads to a p53-mediated mitochondria intrinsic apoptosis through the activation of caspase-3. AGE upregulates sclerostin and RANKL expressions indicating that AGE impairs osteocytes' biological function. Decreased apoptosis and expression of cleaved caspase-3, sclerostin, and RANKL in FOXO1-silenced MLO-Y4 cells provide critical insight as to FOXO1 being an important regulator during this progress. The direct binding of FOXO1 to the caspase-3 promoter provides mechanistic insight into how osteocyte apoptosis is induced, and the binding of FOXO1 to the sclerostin and RANKL provides the mechanism of osteocytes dysfunction. Our results show for the first time that FOXO1 plays as a critical regulator in the AGE-induced osteocyte apoptosis and dysfunction. These results lead to a better understanding of FOXO1's role in diabetes-induced metabolism disorder in the bone tissue and provide an insight mechanism of how diabetes results in an enhanced risk of osteoporosis.

## Data Availability

No data were used to support this study.

## Supplementary Materials

Supplementary Materials The siRNA transfection efficiency by means of knockdown efficiency of FOXO1.
