# Supplementary material for: FOXO1 Mediates Advanced Glycation End Products Induced Mouse Osteocyte-Like MLO-Y4 Cell Apoptosis and Dysfunctions
Source: J Diabetes Res. 2019 Nov 25;2019:6757428. doi: 10.1155/2019/6757428 (PMC6899319; doi:10.1155/2019/6757428)
Supplement: Supplementary Materials — The siRNA transfection efficiency by means of knockdown efficiency of FOXO1. [file 6757428.f1.pptx]

## Slide 1
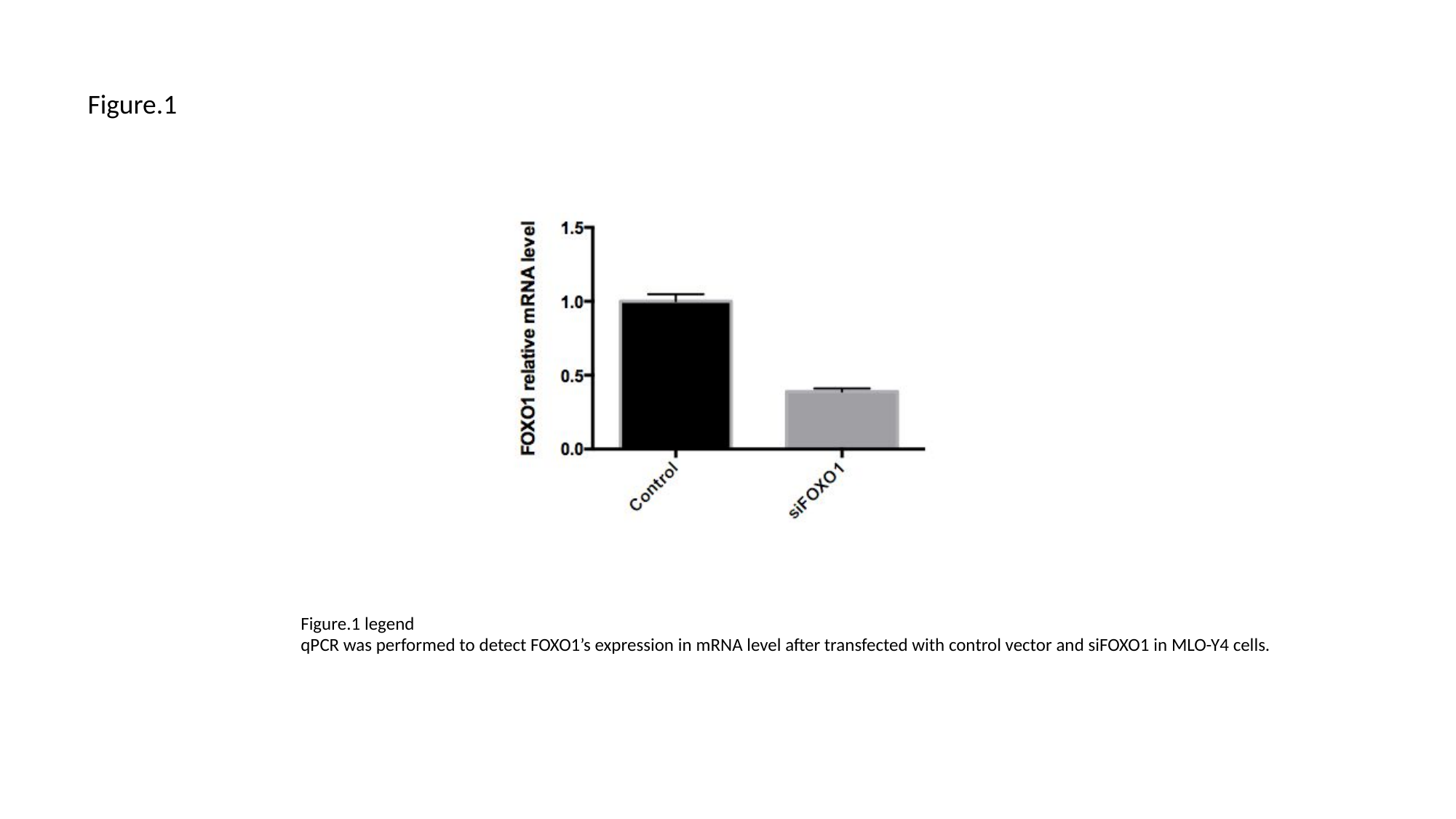

Figure.1
Figure.1 legend
qPCR was performed to detect FOXO1’s expression in mRNA level after transfected with control vector and siFOXO1 in MLO-Y4 cells.
